# The classification of p53 immunohistochemical staining results and patient outcome in ovarian cancer

**DOI:** 10.1038/sj.bjc.6603741

**Published:** 2007-04-17

**Authors:** H Lassus, R Butzow

**Affiliations:** 1 Department of Obstetrics and Gynecology, Helsinki University Central Hospital, Helsinki, Finland; 2 Jorvi Hospital, Department of Obstetrics and Gynecology, Helsinki University Central Hospital, Helsinki, Finland; 3 Department of Pathology, Helsinki University Central Hospital and University of Helsinki, Finland

**Sir**,

We would like to comment on the paper ‘Factors influencing p53 expression in ovarian cancer as a biomarker of clinical outcome in multicentre studies’ by de Graeff *et al* (2006). In this multicentre study, the following classification of p53-immunohistochemical staining results was used: moderate or strong positivity in >50% of tumour cells was considered as aberrant, while other staining patterns (complete lack or weak overall staining or moderate-strong staining in <49% of tumour cells) were classified as normal. In univariate analysis, aberrant p53 was associated with shorter progression-free survival (PFS), but not with overall survival. In multivariate analysis, no independent prognostic value for p53 expression was found. The rationale behind this interpretation of immunohistochemistry is that in-frame point mutations of *p53* alter the conformation of the protein and prolong its biological half-life, thus intensifying the immunohistochemical staining result.

However, this interpretation of p53 immunohistochemical staining leads to false-negative findings (Nenutil *et al*, 2005). Other mechanisms that abolish p53 activity (e.g. homozygous deletion of the gene or truncating mutations) may result in loss of p53 protein and are identified as absence of staining by using sensitive immunohistochemical assay. Null mutations of p53 (nonsense, frameshift and splice-site mutations), which in most cases are associated with negative immunostaining, have been reported in approximately one-fifth of ovarian carcinomas and predict poor patient outcome (Sood *et al*, 1999; Shahin *et al*, 2000). On the basis of this concept, we evaluated the prognostic value of three distinct staining patterns of p53 in serous ovarian carcinoma: ‘normal’ corresponding to heterogeneous positivity that is observed in respective normal tissue (i.e. epithelium of the fallopian tube), ‘excessive’ where the majority (>50%, usually >85%) of tumour cells show homogeneous moderate or strong positivity, and ‘completely negative’, in which no staining is found in any of the carcinoma cells. Both excessive (in 43% of cases) and completely negative (in 16% of cases) p53 staining conferred poor prognosis in serous ovarian carcinoma (Lassus *et al*, 2003). Using this classification, aberrant (excessive or completely negative) p53 expression was a strong and independent prognostic factor for overall survival (OS) in serous ovarian carcinoma. Note that the different staining patterns were distinct, and independent evaluation by two authors was concordant in 97% of the 522 cases (examples of different staining patterns are provided as Supplementary data with the electronic version of the article Lassus *et al*, 2003).

de Graeff *et al* (2006) attempted to validate the classification system we adopted in the serous carcinoma cases of their material. They reported no strong association of p53 with clinical outcome. However, the justification of the conclusion is obscure as, in multivariate analysis of OS, the *P*-value was statistically significant, 0.035. No confidence intervals were presented for the hazard ratios (HR), and there was no information about the proportion of completely p53 negative tumours. Overall, in their material, the association of p53 with poor patient outcome seems stronger with the new classification system (PFS, *P*=0.094, HR 1.48; OS, *P*=0.035, HR 1.70) as compared to the conventional one (PFS, *P*=0.228, HR 1.16; OS, *P*=0.362, HR 1.13), even though the number of cases was much smaller for the analysis using the new classification system as only serous carcinomas were included (*n*=225 *vs n*=476). It is unclear why they chose to present the findings based mainly on the conventional classification of p53 immunostaining.

Both in the study by de Graeff *et al* (2006), as well as our study tumour grade and residual disease remained strong independent prognostic factors. This is consistent with the literature (Friedlander, 1998), and it may be unrealistic to find molecular prognostic markers that would overcome such strong clinical factors.

The molecular mechanisms leading to completely negative p53 immunostaining deserve further studies. In addition to truncating mutations of the gene, other possible mechanisms include homozygous deletion and regulation at transcriptional or translational level. In breast cancer, the p53-related expression fingerprint has been shown as a stronger prognostic factor than mutational status (Miller *et al*, 2005). However, transcriptional fingerprint analyses are laborious and more suitable for scientific purposes. The advantage of immunohistochemistry is that it is easily applicable to routine clinical practice. Immunohistochemistry of p53 downstream genes, especially MDM2, may further improve the accuracy of immunohistochemistry in detecting non-functional p53 (Nenutil *et al*, 2005).
